# Modeling Quorum Sensing Dynamics and Interference on *Escherichia coli*

**DOI:** 10.3389/fmicb.2019.01835

**Published:** 2019-08-20

**Authors:** Carlos E. Torres-Cerna, J. Alejandro Morales, Esteban A. Hernandez-Vargas

**Affiliations:** ^1^Computer Science Department, Universidad de Guadalajara, Guadalajara, Mexico; ^2^Frankfurt Institute for Advanced Studies, Frankfurt, Germany

**Keywords:** AI-2, quorum sensing, cell growth, interference model, *E. coli*, lsr operon, LuxS protein, mathematical modeling

## Abstract

Bacteria control the expression of specific genes by Quorum Sensing (QS). This works using small signaling molecules called Autoinducers (AIs), for example, the Autoinducer-2 (AI-2). In this work, we present a mathematical model that represents the AI-2 dynamics on *Escherichia coli*, which is linked to the cell growth and the *lsr* operon expression. The model is adjusted using experimental data. Our results suggest that the extracellular AI-2 activity level depends on the cell growth rate, and this activity depends on the cell exponential growth phase. The model was adapted to simulate the interference of QS mechanisms in a co-culture of two *E. coli* strains: a wild type strain and a knock out strain that detects AI-2 but does not produce it. Co-culture simulations unveiled two conditions to avoid the QS on the wild strain: when the knock out takes control of the growth medium and overcomes the wild strain, or when is pre-cultured to its mid-exponential phase and then added to the wild strain culture. Model simulations unveiled new insights about the interference of bacterial communication and offer new tools for QS control.

## 1. Introduction

Quorum sensing (QS) is a bacterial communication mechanism used to coordinate cooperative behaviors by producing, releasing, and sensing small signaling molecules called Autoinducers (AIs). Bacteria use these AIs to synchronize specific gene expression within a population (Waters and Bassler, 2005). The QS mechanisms are classified according to the type of AIs, the two of most studied are: the Acyl-homoserine lactone and oligopeptides, produced by Gram-negative and Gram-positive bacteria, respectively (Novick and Geisinger, 2008; Rutherford and Bassler, 2012). These AIs are highly specific, each AI has a cognate receptor in a specific bacterium, and cannot be sensed by other types of bacteria. There is a third type of AIs, the Autoinducer-2 (AI-2), which is produced and sensed by different types of bacteria (Pereira et al., 2013), such characteristic makes them susceptible to interfere in the communication between bacteria that use them as AIs (Xavier and Bassler, 2005a; Laganenka and Sourjik, 2017).

The AI-2 is produced by the LuxS protein, an enzyme involved in the Activated Methyl Cycle (AMC) (De Keersmaecker et al., 2006). Although not all AIs-producing bacteria use them as QS signaling molecules, all the AIs are produced by the LuxS enzyme (Pereira et al., 2013). Despite AIs-2 are produced in the same way in all bacteria by the LuxS enzyme, the signal transduction varies according to each species. Among the bacteria detected so far that use the AI-2 as a signaling molecule, the *Escherichia coli* has attracted the attention of most genetic engineering research. Some *E. coli* pathogenic strains use the AI-2 to regulate two of the most studied phenotypes related to QS: virulence and biofilm formation (Anand and Griffiths, 2003; Li et al., 2007). Other phenotypes that *E. coli* regulates using AI-2 are motility and cell division (Sperandio et al., 2001; González Barrios et al., 2006).

**Figure 1:**
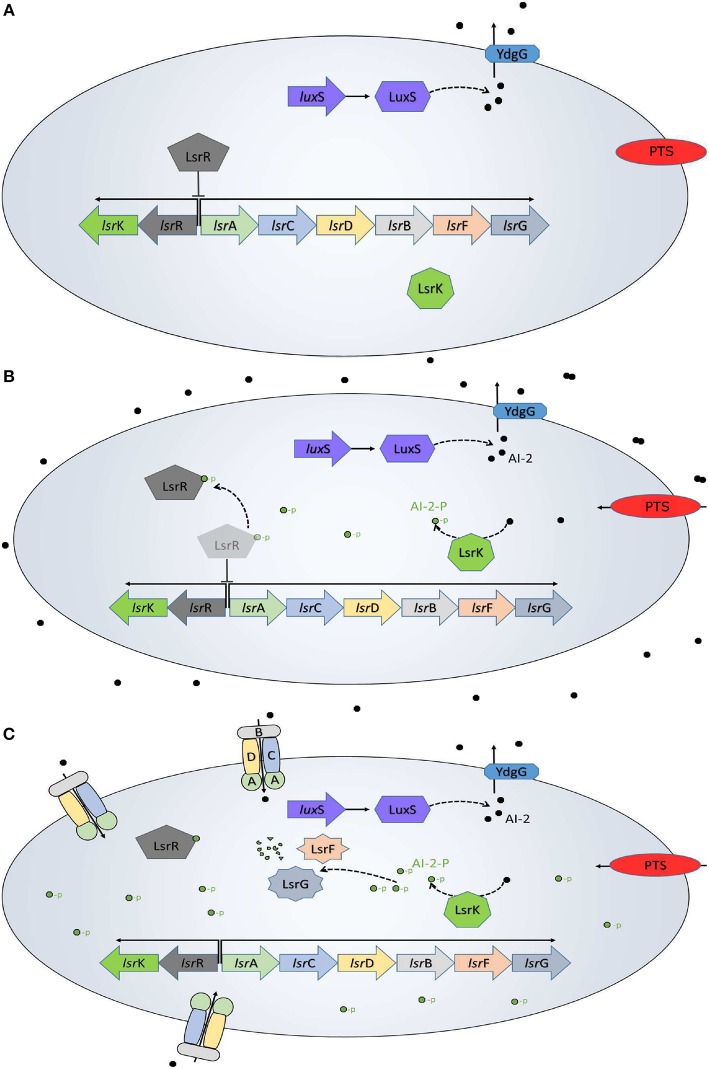
QS in *E. coli*. **(A)** Initially, the AI-2 (black dots) are transported out of the cell, while inside of the bacteria the *lsr* operon is repressed by the LsrR protein. **(B)** The AI-2 accumulation outside of the bacteria increases and the PTS begins the AI-2 uptake. Once back inside of the bacteria, the AI-2 is phosphorylated (green dots) by the LsrK protein and binds to the LsrR protein to de-repress the *lsr* operon. **(C)** The LsrACDB proteins internalize the AI-2 leading a quick depletion of the extracellular AI-2 accumulation. This is a modification from a figure in Pereira et al. (2013).

The QS system in *E. coli* has been well characterized (Xavier and Bassler, 2005a,b; Li et al., 2007; Pereira et al., 2012), and is resumed in Figure 1. AIs-2 are produced as part of the AMC, where the LuxS enzyme catalyzes the 4,5-dihydroxy-2,3-pentanedione and homocysteine. Then, the 4,5-dihydroxy-2,3-pentanedione is spontaneously rearranged into AI-2 (De Keersmaecker et al., 2006) and exported outside of the cells by the membrane protein YdgG. The AI-2 accumulates in the extracellular space until reaches a concentration threshold in a late-exponential growth phase. The phosphoenolpyruvate phosphotransferase system (PTS), a common carbon uptake system in bacteria, was identified as the initial AI-2 consumption pathway and an essential system for the *lsr* operon activation (Pereira et al., 2012). When the AI-2 is internalized back into the cell, it is phosphorylated (AI-2-P) by the kinase LsrK coded in the *lsr*K gene. The AI-2-P binds and represses the repressor protein LsrR, coded on the *lsr*R gene, creating positive feedback and allowing the *lsr* operon expression. Once activated the operon, the AI-2 consumption increases due to the membrane proteins LsrACDB, coded on *lsr*ACDB genes, which decrease the extracellular AI-2 concentration. Additionally to the Lsr operon activation, *E. coli* uses the AI-2 to trigger a set of phenotypes of interest.

Previous works have highlighted the importance of AI-2 dynamics on *E. coli* QS system, including its synthesis, uptake, phosphorylation, and role in *lsr* operon expression (Xavier and Bassler, 2005b; De Keersmaecker et al., 2006; Pereira et al., 2012). In the first *E. coli* QS model, Li et al. developed a stochastic approach to investigate the AI-2 biosynthesis, suggesting additional AI-2 synthesis pathways (Li et al., 2006). Later, Gonzalez Barrios and Achenie (2010) modeled the positive regulation of AI-2 uptake by *lsr* operon activation, keeping the attention on the LsrR protein and its function as *lsr* operon repressor. The idea of the LsrR protein as a “switch” of AI-2 uptake mechanism was presented by Hooshangi and Bentley (2011), they combined their model with experimental data using synthetic AI-2. Additionally, it was modeled the *lsr*ACDB genes knockout, and experimental evidence suggested the existence of a regulatory element parallel to the LsrR protein. Graff and Bentley (2016) presented a detailed model of *lsr* operon expression, which included the molecular interactions between LsrR protein and its binding sites, like the Hooshangi's model, they modeled the LsrR repressor as a “switch” of the QS mechanism.

The AI-2 dynamics could be divided into two main modules for the QS system: the synthesis and the transduction module. The synthesis module is linked with the LuxS enzyme part of the AMC, crucial for the bacterial metabolism. The other module involves the uptake of extracellular AI-2 and the genetic response once the QS is activated. This modularity offers the possibility to develop new approaches of QS control by creating knock out strains that could produce AI-2 without consuming them or consume AI-2 without producing them. In co-cultures with other strains, these knock out strains could lead to overexpression or repression of the *lsr* operon in a wild strain, regulating the QS-related phenotype (Xavier and Bassler, 2005a; Hooshangi and Bentley, 2011; Laganenka and Sourjik, 2017).

In this paper, we propose a mathematical model to capture the AI-2 dynamics on the *E. coli* QS, once adjusted with experimental data, we explore different configurations to simulate the QS interference in co-cultures using two strains: a wild type strain, and a LuxS knock out strain that does not produce AI-2, but sense it (LuxS^−^). Our model is based on three variables used on most experimental works: the *Vibrio harveyi* bacterium as a reporter bacterium to measure the extracellular AI-2 activity (Bassler et al., 1993), the *lsr* operon expression measured by β-galactosidase (β-gal) unit (Koop et al., 1987), and the cell growth. Due to the LuxS protein and the PTS are an inherent part of the bacteria metabolism, the AI-2 and *lsr* operon expression dynamics are modeled as dependent on bacterial growth (Pereira et al., 2012, 2013; Niu et al., 2013). The proposed model describes the AI-2 synthesis, its initial uptake by the PTS, and its uptake after the expression of the LsrACDB proteins. The activation of the *lsr* operon by the PTS and their repression by the LsrR protein are also modeled. Experimental data from Xavier and Bassler (2005a) are used to estimate and evaluate the model parameters. Interference model is a rearrangement of the original model that describes the co-culture of *E. coli* wild type and LuxS^−^ strains. Different configurations of this model were analyzed to exploit the AI-2 consumption rate of the LuxS^−^ in its exponential growth phase, depleting the AI-2 from the extracellular space to avoid the *lsr* operon expression in the wild type strain. Simulation results unveiled key insights about the QS interference, and could lead to investigations of new QS control strategies based on bacterial co-cultures.

## 2. Materials and Methods

###  Quorum Sensing Model in *Escherichia coli*

**Figure 2:**
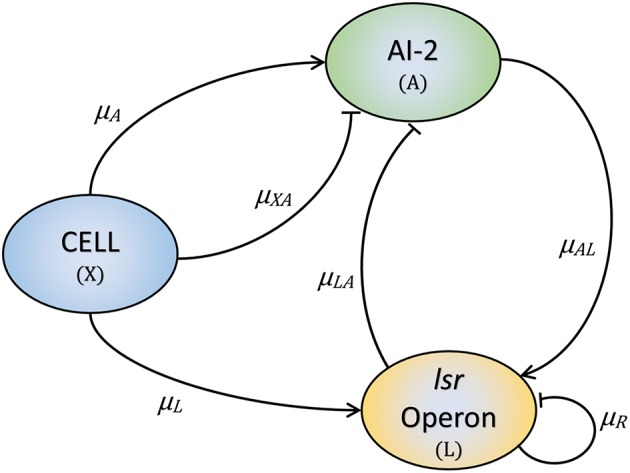
The three key elements involved in the *E. coli* QS mechanism. Bacteria produce and release AI-2 (μ_*A*_) and internalize them through the PTS (μ_*XA*_). Once the *lsr* operon is active by the PTS (μ_*L*_), the LsrACDB proteins internalize the AI-2 (μ_*LA*_), and the AI-2 positively regulates the *lsr*ACDB genes expression (μ_*AL*_). The LsrR protein coded in the *lsr* operon represses the operon expression (μ_*R*_).

The proposed model is developed based on the layout displayed in Figure 2. Our model has three variables: the cell growth (*X*), the *lsr* operon expression (*L*), and the extracellular AI-2 accumulation (*A*). The AIs-2 are produced by *X* and we considered that all are exported outside of the cells, and are internalized by the PTS (Pereira et al., 2012; Niu et al., 2013). *L* is positively regulated by *X* and *A* (Pereira et al., 2012, 2013). Due to AI-2 need to be phosphorylated to activate the *lsr* operon, it is considered that all the extracellular AI-2 are internalized, and phosphorylated back to the bacteria. According to the assumptions above, the mathematical model is composed of the following Ordinary Differential Equations (ODEs):

(1) $$\begin{array}{r} X\left(t\right)=X_{0}+C\;e^{-e^{-B\left(t-M\right)}} \end{array}$$

(2) $$\begin{array}{r} \frac{dA\left(t\right)}{dt}=\mu_{A}-\mu_{XA}-\mu_{LA} \end{array}$$

(3) $$\begin{array}{r} \frac{dL\left(t\right)}{dt}=\mu_{L}+\mu_{AL}-\mu_{R} \end{array}$$

Equation (1) describes the cell growth dynamic, and is represented by a Gompertz function (Zwietering et al., 1990), where *X*_0_ is the initial cell growth, *C* is the asymptote of the function and represents the maximum cell growth, *B* is the slope of the function which represents the growth rate, and *M* is the inflection time. Equation (2) describes the dynamics of *A*. The AI-2 are produced by the cells at rate μ_*A*_, here, we considered that all the AI-2 produced are exported out of the cells. The consumption of *A* by the cells (PTS) is represented by μ_*XA*_. Once the *lsr* operon is expressed, the *A* is depleted by the LsrACDB proteins consumption, this is represented by μ_*LA*_.

The production of the AI-2 depends on the LuxS enzyme as part of the AMC, due to this as an inherent part of the metabolism of the cells, is considered that the AI-2 are produced by the cells. The AI-2 production and extracellular AI-2 accumulation (*A*) is represented by the following Michaelis-Menten function:

(4) $$\begin{array}{r} \mu_{A}=k_{A}\left(\frac{X{\left(t\right)}^{n_{1}}}{X{\left(t\right)}^{n_{1}}+{k_{m1}}^{n_{1}}}\right) \end{array}$$

where *k*_*A*_ is the *A* production velocity, and *k*_*m*1_ is the cell density at which *k*_*A*_ = *k*_*A*_/2. Initially, the cells consume *A* at a basal rate by the PTS, this is modeled using a Monod function as follows:

(5) $$\begin{array}{r} \mu_{XA}=k_{XA}\left(\frac{X{\left(t\right)}^{n_{2}}}{X{\left(t\right)}^{n_{2}}+{k_{m2}}^{n_{2}}}\right)A\left(t\right) \end{array}$$

where *k*_*XA*_ is the PTS uptake rate, and *k*_*m*2_ is the cell growth at half of the maximal PTS consumption rate. When *A* reaches a threshold in a late-exponential growth phase, the *lsr* operon expression is activated leading an increase in the *A* consumption:

(6) $$\begin{array}{r} \mu_{LA}=k_{LA}\left(\frac{X\left(t\right)}{X_{\delta }}\right)\;A\left(t\right)\;L\left(t\right) \end{array}$$

where *k*_*LA*_ is the *A* uptake rate by the LsrACDB proteins and *X*_δ_ is the required cell growth for the increment on the *A* consumption. The Equation (3) represents the expression of the *lsr* operon, initially expressed by the PTS consumption and triggered by the LsrACDB proteins that increase the *A* consumption. Additionally, the *lsr* operon expression is repressed by the LsrR protein. In a similar way that in Equation (5), the *lsr* operon is regulated because the consumption of *A* by the bacteria and is represented by a Monod function as follows:

(7) $$\begin{array}{r} \mu_{L}=k_{L}\left(\frac{X{\left(t\right)}^{n_{3}}}{X{\left(t\right)}^{n_{3}}+{k_{m3}}^{n_{3}}}\right)A\left(t\right) \end{array}$$

where *k*_*L*_ is the operon expression rate. *k*_*m*3_ is the cell growth at half of the maximal PTS consumption rate. The expression of the *lsr* operon is triggered by the LsrACDB proteins, this dynamic is presented as follows:

(8) $$\begin{array}{r} \mu_{AL}=k_{AL}\left(\frac{X\left(t\right)}{X_{\delta }}\right)\;A\left(t\right)\;L\left(t\right) \end{array}$$

where *k*_*AL*_ is the *lsr* operon expression rate. Finally, the *lsr* operon is repressed by the LsrR protein at rate *k*_*R*_, hence:

(9) $$\begin{array}{r} \mu_{R}=k_{R}\;L\left(t\right). \end{array}$$

### 2.1. Parameter Estimation

Parametric estimation of mathematical models can be understood as the search of values for the parameters set θ that minimize the difference between the model outcome *ȳ*_*i*_ and the experimental data *y*_*i*_ as close to zero as possible. The Sum of the Square of Weighed Residues (*SSWR*) has been used in other works as cost function (Patwardhan and Srivastava, 2004; Khanna and Srivastava, 2006; Torres-Cerna et al., 2017). This function allows evaluating the difference of all variables in the same function, adding weights to normalize them. It is defined as follows:

(10) $$\begin{array}{r} SSWR\left(\theta \right)=\overset{m}{\underset{j=1}{\sum }}\overset{n}{\underset{i=1}{\sum }}{\left(\frac{y_{i}^{j}-{ȳ}_{i}^{j}}{max\left(y^{j}\right)}\right)}^{2} \end{array}$$

where *j* and *i* represents the number of variables and experimental data points, respectively, *y* is the set of experimental data points, and *ȳ* is the model outcome. Since the ODE integration routine requires dense data sets at different times depending on an adaptive stepsize, the inputs for each estimation are approximated by linear interpolation. The minimization of Equation (10) implies a non-linear optimization problem with several variables that can be solved using a global optimization algorithm. In this work, we use the Differential Evolution (DE) algorithm (Storn and Price, 1997) to estimate the best parameter values set.

### 2.2. Parameter Identifiability

Parameter identifiability plays an important role in mathematical modeling, the identifiability analysis of unknown parameters of a non-linear mathematical model is not a trivial task (Miao et al., 2011). To analyze the identifiability of each parameter in Equations (1–3), we use the profile likelihood method proposed by Raue et al. (2009), that additionally explores the structural identifiability. Briefly, the method consists of defining a set of values centered at the optimized value for each parameter, and minimize the SSWR re-optimizing the remaining parameters. Further details of parameter fitting procedures and identifiability can be found in Nguyen et al. (2016) and Hernandez-Vargas (2019).

### 2.3. Parameter Uncertainty

Data variability is an inherent characteristic of the biological system because of their stochastic nature. Furthermore, the measuring methods can generally add noise to the experimental data. A statistic method to measure parameters accuracy is the weighted bootstrap method (Xue et al., 2010). This method assigns a vector of exponentially distributed random weights, with mean and variance one, to the cost function. After bootstrap, 95% of the confidence interval of each parameter was computed using the 2.5 and 97.5% quantiles. Furthermore, the parameter dependency can be analyzed based on the bootstrap results.

### 2.4. Interference Model

The use of mutant strains of *E. coli* is a common practice to understand the QS mechanism and identify the key elements (Wang et al., 2005; Xavier and Bassler, 2005b; Hooshangi and Bentley, 2011; Pereira et al., 2012). In this work, we simulated the co-culture of two strains of *E. coli*: a wild type strain and a luxS knock out strain (luxS^−^), this strain does not produce AIs but can sense them by the PTS and the LsrACDB proteins. Because the bacteria in co-culture sharing the growth medium, neither wild or knock out strains can grow at the maximum bacterial concentration (*C*). Instead, assuming that *C* is the highest possible bacterial concentration, it is considered that both bacteria share it. Different values of maximum concentration for both strains are simulated in order to understand the effects of co-cultures of wild and knock out strains. For these simulations, the model was modified adding a second growth function for the LuxS^−^ strain.

(11) $$\begin{array}{r} X_{ko}\left(t\right)=X_{0}+C_{ko}\;e^{-e^{-B\left(t-M\right)}} \end{array}$$

where *C*_*ko*_ is the maximum bacterial concentration for the LuxS^−^ strain, and represents a percentage of *C*. Additionally, the growth function for the wild strain is a modification of (1) as follows:

(12) $$\begin{array}{r} X\left(t\right)=X_{0}+C_{w}\;e^{-e^{-B\left(t-M\right)}} \end{array}$$

where *C*_*w*_ is the maximum bacterial concentration for the wild strain, and represents a percentage of *C*, then *C*_*ko*_ + *C*_*w*_ = *C*. The consumption of AI-2 by the LuxS^−^ is also added to the model, by modifying (2) as follows.

(13) $$\begin{array}{r} \frac{dA\left(t\right)}{dt}=\mu_{A}-\left(\mu_{XA}+\mu_{XA_{ko}}+\mu_{LA}+\mu_{LA_{ko}}\right) \end{array}$$

where μ_*XA*_*ko*__ and μ_*LA*_*ko*__ are modifications of (5) and (6), respectively. This writes as follows:

(14) $$\begin{array}{r} \mu_{XA_{ko}}=k_{XA}\left(\frac{X_{ko}{\left(t\right)}^{n_{2}}}{X_{ko}{\left(t\right)}^{n_{2}}+{k_{m2}}^{n_{2}}}\right)A\left(t\right) \end{array}$$

(15) $$\begin{array}{r} \mu_{LA_{ko}}=k_{LA}\left(\frac{X_{ko}\left(t\right)}{X_{\delta }}\right)\;A\left(t\right)\;L\left(t\right) \end{array}$$

## 3. Results

Parameter fitting was assessed using the experimental data in the work of Xavier and Bassler (2005a), from this work we can use the three variables that we consider important in the *E. coli* QS dynamics: the extracellular AI-2 activity, the *lsr* operon expression, and the cell growth. The extracellular AI-2 activity on *E. coli* was analyzed indirectly by measuring the bioluminescence produced by the reporter bacterium *Vibrio harveyi* (Bassler et al., 1993), the *lsr* operon expression was analyzed by measuring the β-galactosidase units (Koop et al., 1987), and the cell growth is measured by the optical density. The bacterial growth, extracellular AI-2 activity, and *lsr* operon expression data were obtained from figures using the Plot Digitizer software (Huwaldt and Steinhorst, 2013). The number of experimental data points for cell growth and extracellular AI-2 accumulation are the same, unlike the *lsr* operon expression which has fewer data points. Since the minimization of *SSWR* requires the same number of experimental data points for each variable, the experimental values were approximated by linear interpolation. Additionally, due to the growth function is independent on others equations, the parameters in Equation (1) were estimated separately and their best-estimated values were fixed for parameter estimations of Equations (2) and (3), reducing the model complexity and computational time. The best-estimated values of parameters were used to analyze the profile likelihood.

**Figure 3:**
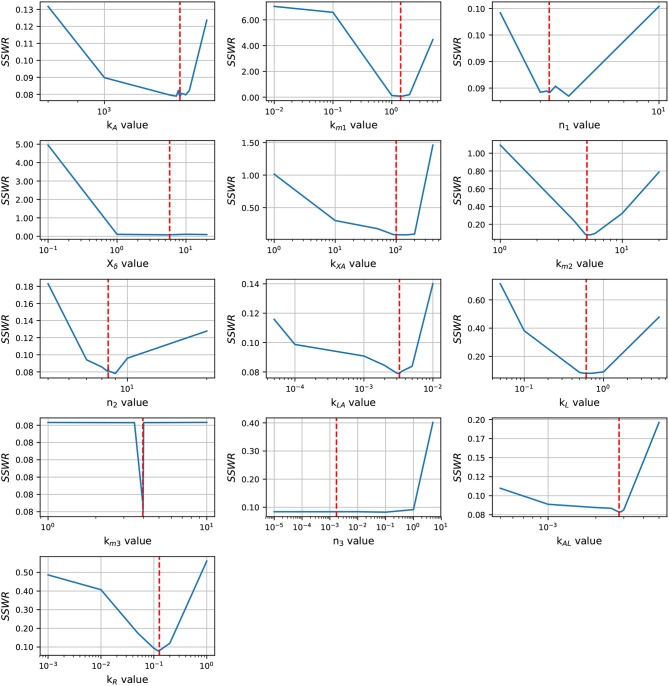
Profile likelihood. Parameter identifiability is analyzed using the profile likelihood. A parameter is identifiable if there is a parameter value that minimizes the cost function, the concave shape in graphics shows that the cost function can be minimized. The vertical red line represents the best parameters value.

Before an exhaustive estimation of the model parameters, we analyzed the parameter identifiability using the profile likelihood method (Raue et al., 2009). The results in Figure 3 shows a concave shape in most of parameter graphics, denoting a finite set of values which can minimize the *SSWR*, but some parameters show a flattens profile. The profile likelihood graphic of *X*_δ_ tends to flatten to the right, biologically, the *lsr* operon expression increases as the cell growth approaches to *X*_δ_, this value must be close to the cell growth in the late-exponential phase. The likelihood graphic for parameter *n*_3_ tends to flatten close to zero, which means that can take any value arbitrary small and do not enhance the model fit. According with the profile likelihood graphics, parameters *n*_3_ and *X*_δ_ are practically non-identifiable (Raue et al., 2009).

**Figure 4:**
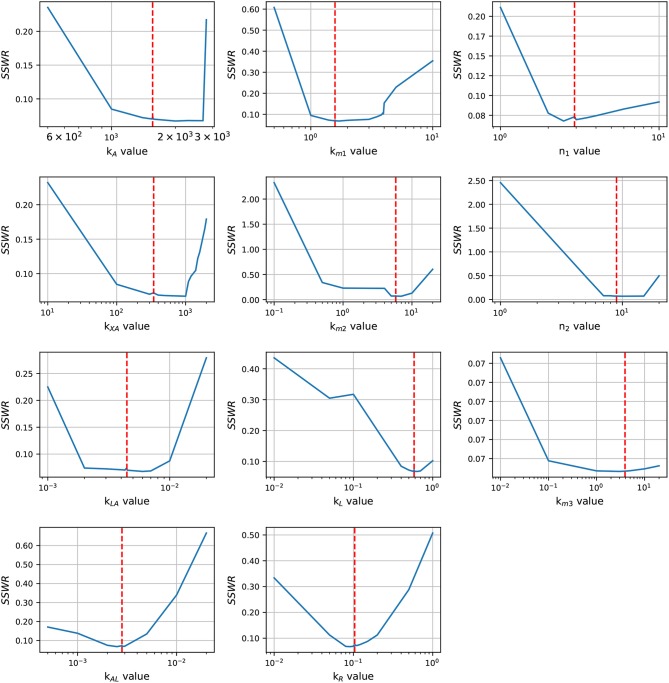
Profile likelihood. After fixing parameters *X*_δ_ and *n*_3_, parameter identifiability is re-analyzed for the remaining parameters using the profile likelihood. The vertical red line represents the best parameters value.

In order to enhance the model fit and considering the profile likelihood graphics, additional estimations were made fixing parameters *n*_3_ and *X*_δ_. Based on the profile likelihood graphics, the parameter *n*_3_ was fixed at *n*_3_ = 0.001722, and based on the profile likelihood and the biological restrictions due to *X*_δ_ should be below the maximum cell growth and close to the growth at late-exponential growth phase, was fixed at *X*_δ_ = 5.7953. These values were taken from the set of parameters used to analyze the profile likelihood. After fixing these parameters, the profile likelihood for the remaining parameters was analyzed again in order to identify changes on their identifiability. Despite of the parameter identifiability changed after fixing *n*_3_ and *X*_δ_, the profile likelihood plots in Figure 4 show that fixing parameters *k*_*m*3_ and *X*_δ_, the remaining parameters are still identifiable. The profile likelihood plot of *k*_*m*3_ shows that it has a small influence on the model fitness, this could imply that the PTS consume of AI-2 has a weak effect on the *lsr* operon expression.

**Figure 5:**
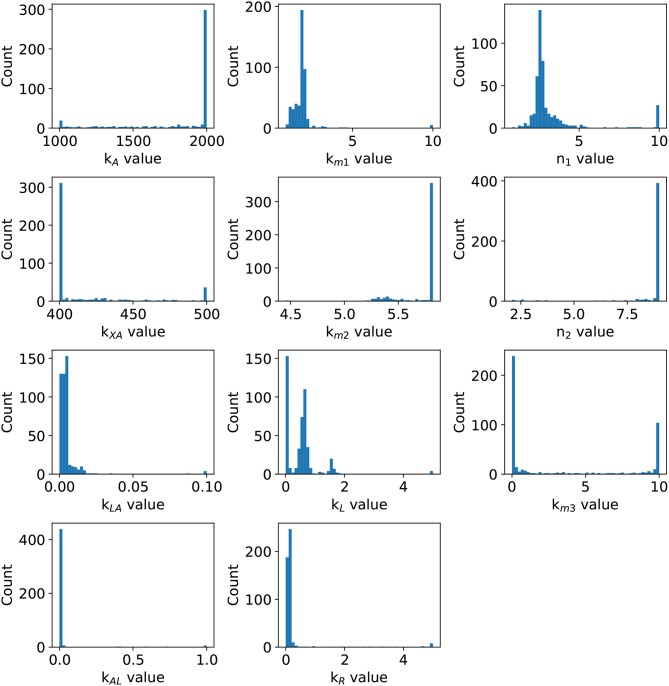
Parameter distribution. After 500 weighted bootstrap repetitions, the distribution of parameters is depicted.

**Figure 6:**
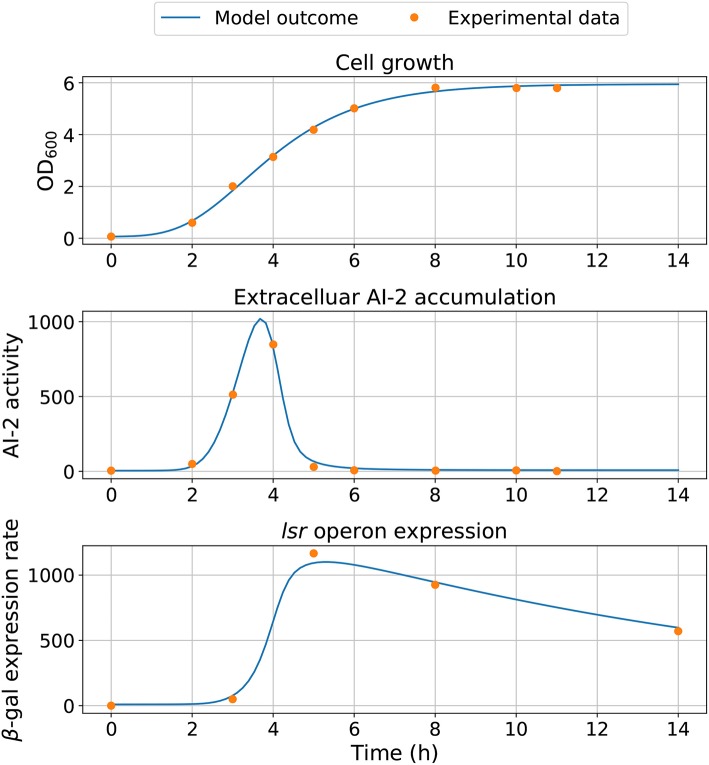
Model simulation. The experimental data are represented by close circles and model outcome in a continuous line.

Statistical insights of the parameter fitting can be acquired from the bootstrap method (Xue et al., 2010). After 500 bootstrap reproductions, the 95% confidence interval for each parameter was calculated using the 2.5 and 97.5% quantiles, the confidence interval can be seen in Table 1. The parameters distribution are depicted in Figure 5. After fixing parameters *n*_3_ and *X*_δ_, the model outcome is presented in Figure 6, the experimental data (Xavier and Bassler, 2005a) is presented in close circles and the model outcome in continuous line, the set of model parameters are presented in Table 1.

**Table 1:** Parameters values.

**Parameter**	**Best fit**	**Confidence interval**	**Description**
*X*_0_^*^ (*OD*_600_)	0.064		Initial cell concentration
C^*^ (*OD*_600_)	5.8828		Maximum bacterial concentration
*B* (*t*^−1^)	0.6384		Cell growth rate
*M* (*t*)	3.2823		Cell growth inflection time
*k*_*A*_ ($\frac{OD_{490}}{t}$)	1, 561.68	1, 000−2, 000	AI-2 velocity production
*k*_*m*1_ (*OD*_600_)	1.5793	0.8491−10	the cell growth at half of *k*_*A*_
*X*_δ_^**^ (*OD*_600_)	5.7953		Bacterial concentration at the stationary phase
*k*_*XA*_ (*t*^−1^)	343.98	400−500	AI-2 uptake rate by PTS
*k*_*m*2_ (*OD*_600_)	5.8205	4.4554−5.8211	Cell growth at half of *k*_*XA*_
*k*_*LA*_ (β−*gal*·*t*^−1^)	0.0044	0.0001−0.0999	AI-2 uptake rate by LsrACDB proteins
*k*_*m*3_ (*OD*_600_)	3.9128	0.001−10	Cell growth at half of *k*_*L*_
*k*_*L*_ ($\frac{\beta -gal}{OD_{490}\cdot t}$)	0.5825	0.1−5	Operon expression rate
*k*_*AL*_ (${\left(OD_{490}\cdot t\right)}^{-1}$)	0.0028	0.0001−1	*lsr*ACDB genes expression rate
*k*_*R*_ (*t*^−1^)	0.1037	0.001−5	*lsr*R gene expression rate
*n*_1_	2.9302	0.8096−10	Exponent for adjusting curves
*n*_2_	8.9542	2.0857−9	Exponent for adjusting curves
*n*_3_^**^	0.0017		Exponent for adjusting curves

The symbol ^*^ means that parameters were fixed according to data in Xavier and Bassler (2005a). Symbol ^**^ means that parameters were fixed based on the profile likelihood graphics. The confidence interval after 500 bootstrap repetitions was calculated within 2.5 and 97.5% quantiles, the confidence interval for parameters in Equation (1) were not calculated.

**Figure 7:**
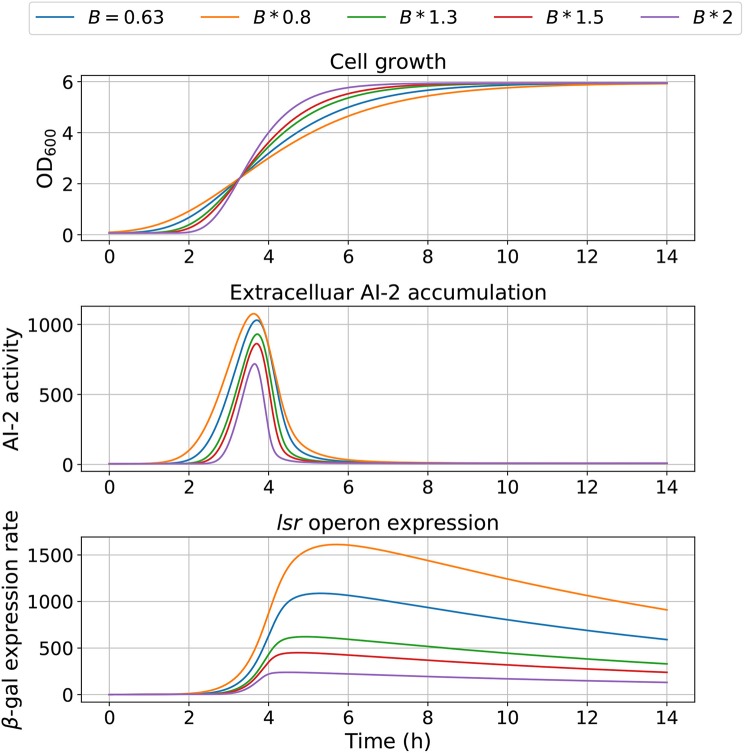
Model outcome at different growth rates. Parameter *B* was modified to change the bacterial growth behavior by multiplying the optimal value *B* = 0.63 by different factors.

**Figure 8:**
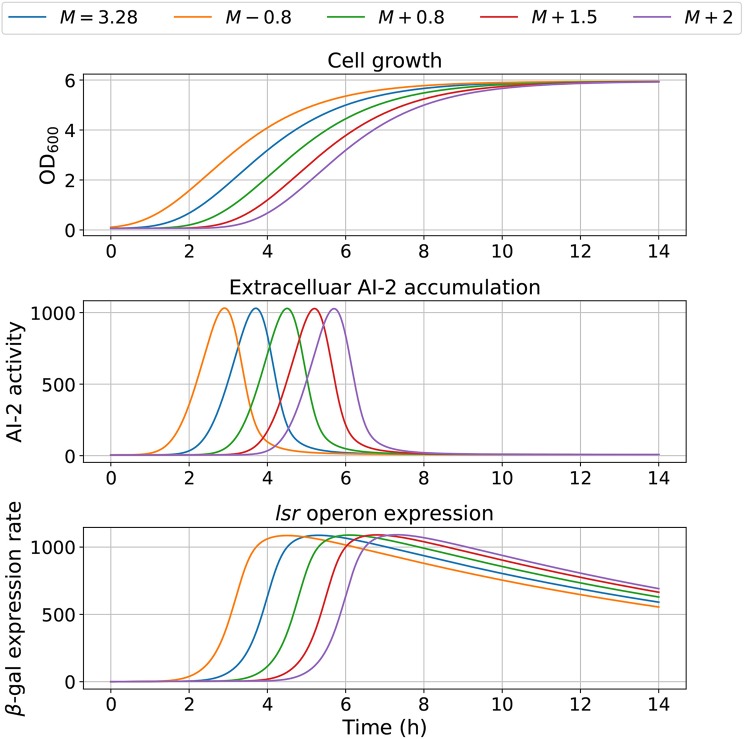
Model outcome with different inflection time in growth. Parameter *M* was modified to change the bacterial growth behavior, by increasing or decreasing its optimal value *M* = 3.28.

**Figure 9:**
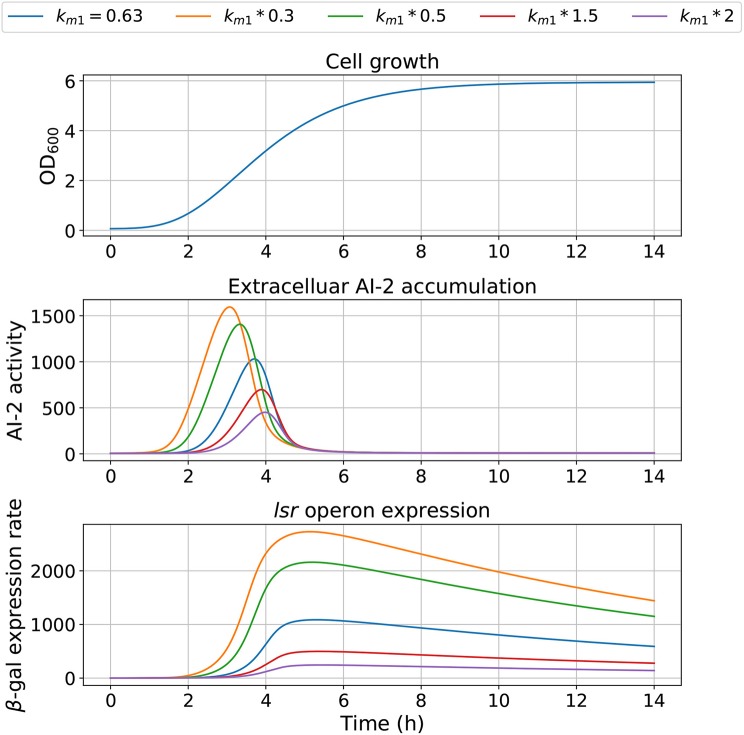
Model outcome with values for *k*_*m*1_. Parameter *k*_*m*1_ was modified to change the bacterial growth behavior, changing the bacterial concentration for trigger the AI-2 extracellular concentration.

The model dependency on cell growth is analyzed using different growth rates and inflection time in simulations, the results are depicted in Figures 7, 8, respectively. The simulations reveal that the cell growth at an initial exponential phase (*X* ≈ 0.7) could trigger an exponential increase in the extracellular AI-2 accumulation. The cell growth rate also affects the maximal extracellular AI-2 accumulation (Figure 7), which reaches its maximum in the half-exponential growth phase, this means that the exponential growth phase is where the AI-2 has its maximum production. When the cell growth rate is large, the exponential growth phase is brief, then the time of AI-2 maximum production is also of short duration. Additionally, the *lsr* operon expression depends on the extracellular AI-2 accumulation. The inflection time does not affect the extracellular AI-2 accumulation (Figure 8), but this affects the time of maximal production and depletion. In both figures, the cell growth that triggers the extracellular AI-2 accumulation is the same. Furthermore, the cell density at the extracellular AI-2 accumulation increases exponentially is controlled by *k*_*m*1_, this can be seen in Figure 9, where the time of the exponential growth phase was modified by changing the parameter *k*_*m*1_. In a similar way like increasing the growth rate, increasing *k*_*m*1_ decreases the maximum extracellular AI-2 accumulation, also due to the window of cell maximum production is shorter.

Results of the interference simulations are shown in Figures 11, 12. In former figure, we simulate a wild strain maximum concentration of 10, 30, 50, 70, and 90% of *C*, and LuxS^−^ strain maximum concentration of 90, 70, 50, 30, and 10% of *C*, respectively. When both strains grow equally (50/50%), the operon expression of wild strain is three times bigger than in culture alone. Considering that both strains grow equally, Figure 12 presents the knock out strain was pre-culture with different times and added to the co-culture with the wild strain. In the mid-exponential phase (from the hour three or after), the consumption of AI-2 by the PTS is enough to deplete the extracellular AI-2 and avoid the *lsr* operon expression.

## 4. Discussion

The identifiability analysis shows that parameter *k*_*m*3_ has a small influence on the model fitness, this could be attributed to the effect of the initial uptake by the PTS has on the extracellular AI-2 accumulation is not significant in comparison with the effect of the LsrACDB proteins on the *lsr* operon expression. Despite the PTS is important to the *lsr* operon expression (Pereira et al., 2012), its influence is not included in the mathematical models of *E. coli* QS, and when is included its influence is not significant (Hooshangi and Bentley, 2011).

The AI-2 synthesis and PTS are two important elements involved in the QS mechanism (De Keersmaecker et al., 2006; Pereira et al., 2012), both elements are inherent to the bacteria metabolism (Doherty et al., 2002; De Keersmaecker et al., 2006). The role that bacterial growth plays for AI-2 synthesis was shown by Wang et al. (2005) who unveiled that the AI-2 synthesis and uptake by PTS are subject to the metabolite repression. In addition, they proved that adding or removing glucose from the growth medium affects the AI-2 synthesis. Additionally, experimental works on *E. coli* QS system, show that the *lsr* operon expression is triggered by the extracellular AI-2 concentration in the late-exponential phase (Xavier and Bassler, 2005a,b; Li et al., 2007; Pereira et al., 2012). There is a relation between the bacterial concentration and the *lsr* operon expression, also linked to the AI-2 accumulation.

**Figure 10:**
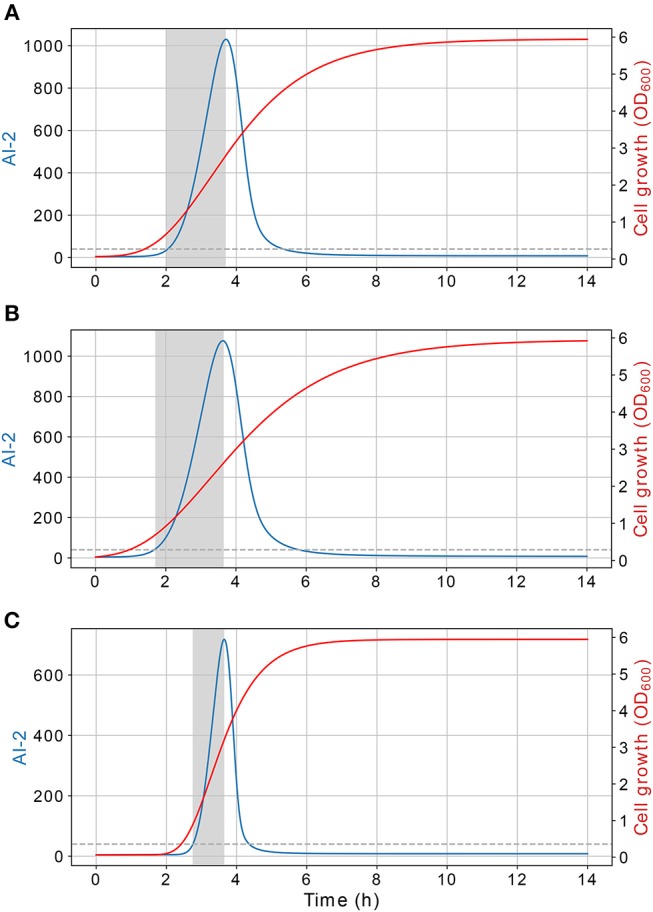
Extracellular AI-2 activity. Time of the exponential growth of the extracellular AI-2 activity depends on the cell growth rate, this relation is plotted using different cell growth rates, **(A)** *B* = 0.63, **(B)** *B* = 0.5, **(C)** *B* = 1.26. The extracellular AI-2 activity grows exponentially during the cell exponential growth phase, in a late-exponential growth phase the extracellular accumulation is depleted due to the action of the LsrACDB proteins.

According to the simulations, the dynamics of the proposed model are dependent on the cell growth, which controls both, the extracellular AI-2 accumulation and the *lsr* operon expression. The former increases exponentially on the cell exponential growth phase, and start to decrease in the late-exponential growth phase. The cell growth rate has an effect on the extracellular AI-2 accumulation and increases o decreases the time of the exponential growth phase, the AI-2 accumulation is proportional to the time that the cells spend on the exponential growth phase before the AI-2 consumption rate may overcome the AI-2 production rate (Figure 10). This occurs because of the AI-2 de-repress the *lsr* operon, allowing the LsrACDB genes expression when the cells reach a certain concentration, increasing the AI-2 consumption (Pereira et al., 2013).

Furthermore, the *lsr* operon expression level is proportional to the AI-2 extracellular accumulation at the activation moment, the more AI-2 activity there is in the extracellular environment, the greater the expression of the *lsr* operon, and the AI-2 consumption. As some experiments have shown, *E. coli* can increase its QS-related phenotypes when they are grown in co-cultures with bacteria that produces AI-2 (Xavier and Bassler, 2005a; Laganenka and Sourjik, 2017). The *lsr* operon give to *E. coli* the advantage to increase the consumption of extracellular AI-2, and therefore promote the phenotypes related to the QS, like biofilm formation or virulence.

Previous experiments have shown the importance of the culture in the AI-2 production, like the type (Jackson et al., 2002; Kim et al., 2015), and the carbon availability (Surette et al., 1999; DeLisa et al., 2001; Wang et al., 2005), and how this affects the phenotypes related to the QS (Oh et al., 2007; Yoon and Sofos, 2008; Niu et al., 2013). Approaches to reduce or inhibit the QS-related phenotypes add agents that antagonize the QS activity, some of these agents are: lactobacillus (Park et al., 2014), cannabinoids (Soni et al., 2015), lactic acids (Almasoud et al., 2016), and honey (Lee et al., 2011). These numerical results could lead the motivation for the study of a new approach for QS control based on the growth media.

**Figure 11:**
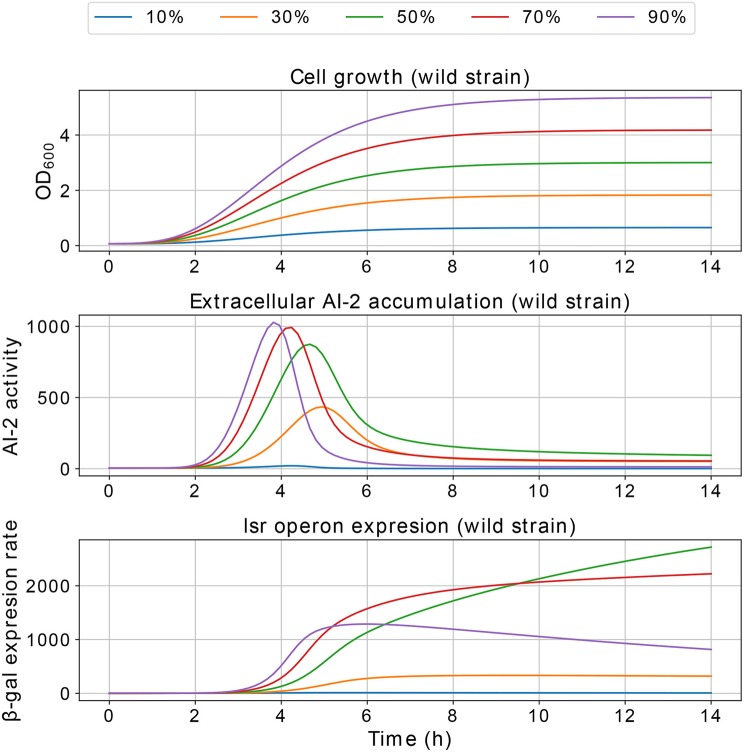
Simulations of *E. coli* co-culture. Simulation of co-cultures with *E. coli* wild and LuxS^−^ strains, the percentages indicate the wild strain growth percentage with respect to *C*, the growth of the LuxS^−^ strain is complementary.

**Figure 12:**
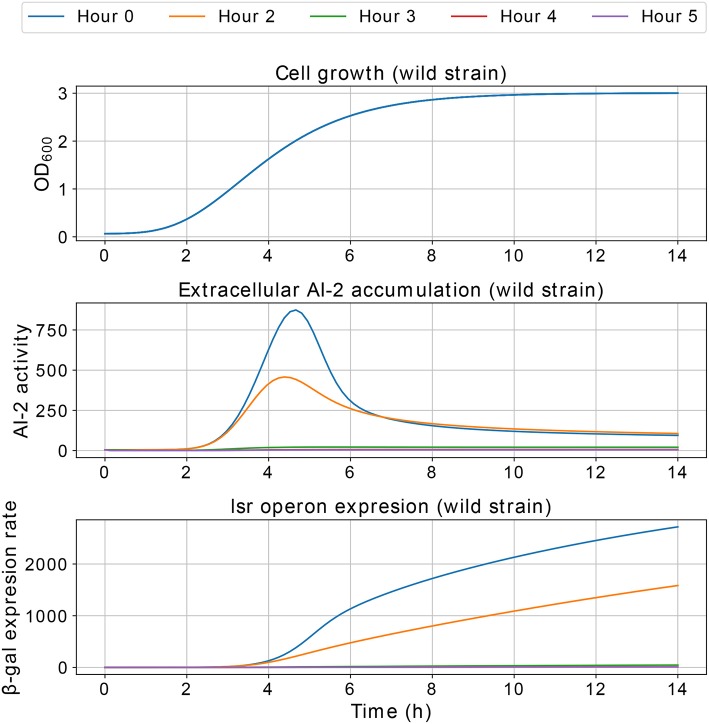
Simulations of *E. coli* co-culture. The LuxS^−^ strain was simulated in a pre-culture and added to the culture with the wild strain at 0, 2, 3, 4, and 5 h of pre-culture, and both strains share equally the growth medium.

The simulations of co-cultures of wild and LuxS^−^ strains show a reduction in the *lsr* operon expression due to the uptake of AI-2 by the LuxS^−^ strain. When the LuxS^−^ strain levels overcome the concentration of the wild strain (blue and orange lines in Figure 11), there is not enough production of AI-2 to activate the *lsr* operon. On the other hand, when both strains grow equally, or the wild type levels overcome the concentration of the LuxS^−^strain, the *lsr* operon expression is bigger than when wild strain grows alone (Figure 6). This overexpression is mainly due to the consumption by the PTS by the wild strain, because the influence of the LsrACDB proteins is maximum when the bacteria reach a value close to the concentration on the stationary phase (*C*). Assuming that both strains growth equally in co-culture, we simulated a pre-culture of the LuxS^−^ strain that was added to the wild strain culture, a pre-culture of LuxS^−^ strain increase its possibilities of consuming the AIs faster than the wild strain and avoid the wild strain *lsr* operon expression. The results depicted in Figure 12, show that a pre-culture starting at 3 h or after of a LuxS^−^ strain, added to a wild strain in a co-culture, can mitigate its *lsr* operon expression.

The simulations of the interference model offer a new alternative to control the phenotypes related to the QS in co-cultures. Some studies have studied the variations on the QS-related phenotypes when two bacteria grow in a co-culture (Xavier and Bassler, 2005a; Roy et al., 2010; Laganenka and Sourjik, 2017), others have studied the impact of gene knock out in QS (Xavier and Bassler, 2005b; González Barrios et al., 2006; Hooshangi and Bentley, 2011). According to our simulations, using knock out bacteria that compete for the extracellular AIs and avoid the expression of genes related to QS can be an approach to control the QS-related phenotypes.

## 5. Conclusions

In this paper, we presented a mathematical model that captures the relationship between AI-2 dynamics, the *lsr* operon expression, and the cellular growth, three key elements involved in *E. coli* QS system and variables normally used on experimental works. The dependency of the extracellular AI-2 activity and the *lsr* operon expression on the cell growth was highlighted, drawing attention to the cellular growth rate and propose the culture medium as an option to control the QS system on *E. coli*. Our simulations suggest that cell growth controls the levels of extracellular AI-2 concentration, and in turns, this controls the levels of *lsr* operon expression.

The AI-2 synthesis and PTS are two important elements involved in QS mechanism (De Keersmaecker et al., 2006; Pereira et al., 2012), both elements are inherent to the bacteria metabolism (Doherty et al., 2002; De Keersmaecker et al., 2006). The role that bacterial growth plays for AI-2 synthesis was shown by Wang et al. (2005) who unveiled that the AI-2 synthesis and uptake by PTS are subject to the metabolite repression. In addition, they proved that adding or removing glucose from the growth medium affects the AI-2 synthesis. Additionally, experimental studies on *E. coli* QS system show that the *lsr* operon expression is triggered by the extracellular AI-2 accumulation in the late-exponential phase (Xavier and Bassler, 2005a,b; Li et al., 2007; Pereira et al., 2012).

The identifiability analysis of *k*_*m*3_ may suggest that the influence of the PTS system on the global QS response is not significant. Nevertheless, additional simulations of the interference model suggest the PTS influence is important in the exponential growth phase. Our simulations suggest that its influence could avoid the expression of *lsr* operon, this can lead to QS-related phenotypes not being expressed. These observations could be performed *in-vitro* in order to confirm the simulations and pave the road to new approaches for QS control.

## Data Availability

The raw data supporting the conclusions of this manuscript will be made available by the authors, without undue reservation, to any qualified researcher.

## Author Contributions

CT-C developed the model and performed the simulations. EH-V envisaged and supervised the project. CT-C, JM, and EH-V discussed and wrote the paper.

### Conflict of Interest Statement

The authors declare that the research was conducted in the absence of any commercial or financial relationships that could be construed as a potential conflict of interest.
